# Germinated Andean Lupin Whole Flour as a Partial Soy Protein Isolate Substitute for the Development of High-Moisture Extruded Meat Analogues: Chemometric Evaluation of Technological Properties and Nutritional and Functional Characterization

**DOI:** 10.3390/foods15152633

**Published:** 2026-07-27

**Authors:** Luz María Paucar-Menacho, Anggie Verona-Ruiz, Alicia Lavado-Cruz, Williams Esteward Castillo-Martínez, Wilson Daniel Simpalo-Lopez, Grimaldo Quispe-Santivañez, John Gonzales-Capcha, Wenceslao T. Medina, Nathalia de Andrade Neves, Marcio Schmiele

**Affiliations:** 1 Departamento Académico de Agroindustria y Agronomía, Facultad de Ingeniería, Universidad Nacional del Santa, Nuevo Chimbote 02712, Peru; 2023720002@uns.edu.pe (A.V.-R.); 2023720003@uns.edu.pe (A.L.-C.); wcastillo@uns.edu.pe (W.E.C.-M.); wsimpalol@uns.edu.pe (W.D.S.-L.); jkgonzales@uns.edu.pe (J.G.-C.); 2 Escuela Profesional de Ingeniería Agroindustrial, Universidad Nacional Autónoma Altoandina de Tarma, Junín 12731, Peru; gquispe@unaat.edu.pe; 3 Departamento Académico de Ingeniería Agroindustrial, Facultad de Ciencias Agrarias, Universidad Nacional del Altiplano de Puno, Puno 21001, Peru; wtmedina@unap.edu.pe; 4 Laboratório de Pesquisa em Bioativos de Plantas (FITOLAB), Departamento de Agronomia, Faculdade de Ciências Agrárias (FCA), Universidade Federal dos Vales do Jequitinhonha e Mucuri (UFVJM), Diamantina 39100-000, Brazil; nathalia.neves@ufvjm.edu.br; 5 Laboratório Integrado de Cereais e Lipídeos (LICEL), Instituto de Ciência e Tecnologia (ICT), Universidade Federal dos Vales do Jequitinhonha e Mucuri (UFVJM), Diamantina 39100-000, Brazil

**Keywords:** alternative proteins, amino acid composition, Andean legumes, circular bioeconomy, fibrous structure, plant-based foods, protein quality, cooking properties

## Abstract

Germinated Andean lupin whole flour (GAL) is rich in protein, dietary fiber, essential amino acids, and bioactive compounds, representing a promising alternative for the development of sustainable plant-based foods. This study investigated the feasibility of partially replacing soy protein isolate (SPI) with GAL in high-moisture extruded meat analogues. A central composite design was applied to evaluate the effects of the GAL ratio (0:100–50:50) and feed moisture content (50–70%) on the technological properties of the extrudates. The Response Surface Methodology was used to model and optimize the process. The incorporation of GAL significantly affected the (*p* < 0.10) water solubility index (WSI), oil absorption capacity (OAC), cooking loss (CL), yellowness (b*), cohesiveness, and adhesiveness, generating predictive models with satisfactory goodness-of-fit (R^2^ > 0.75). Increasing GAL levels increased the WSI from 6.21 to 18.79% and cooking loss from 1.01 to 3.84%, while reducing OAC from 239.42 to 166.57%, indicating substantial modifications in matrix organization and hydration behavior. Numerical optimization identified an optimal formulation containing 12% GAL, 88% SPI, and 65.5% feed moisture, with a desirability of 77.82%. Model validation showed relative deviations lower than 10% between predicted and experimental values. The optimized meat analogue exhibited high protein content (84.44%), favorable techno-functional properties, and improved nutritional quality, with higher levels of branched-chain amino acids (17.34 g·100 g^−1^ protein), essential amino acids (33.32 g·100 g^−1^ protein), and in vitro protein digestibility (90.1%) compared with the control formulation. Multivariate analyses confirmed that phenylalanine, histidine, methionine, and leucine were the main variables that discriminated between the protein sources and the extruded products. Overall, GAL demonstrated strong potential as a sustainable functional ingredient to produce nutritionally enhanced high-moisture meat analogues.

## 1. Introduction

The growing demand for alternative protein sources has accelerated the development of plant-based meat analogues, driven by increasing concerns regarding environmental sustainability, human health, and global food security. In this context, grain legumes have emerged as highly promising ingredients due to their favorable nutritional composition, technological functionality, and lower environmental footprint compared with animal-derived proteins. Among these crops, Andean lupin (*Lupinus mutabilis* Sweet), also known as *tarwi*, *chocho*, *tremoço*, and *altramuz*, is an underutilized legume native to the Andean region that has attracted growing scientific interest because of its exceptionally high protein content, ranging from 32.0 to 52.6 g·100 g^−1^ dry matter, as well as its balanced amino acid profile and valuable techno-functional properties for food applications [1,2].

Several structuring technologies have been employed to produce plant-based meat analogues, including electrospinning, shear-cell processing, three-dimensional (3D) food printing, and thermoplastic extrusion. Among these approaches, high-moisture extrusion remains the most widely adopted industrial technology due to its ability to generate anisotropic and fibrous structures that closely resemble the texture and appearance of animal muscle tissue. During high-moisture extrusion, plant proteins are subjected to intense thermal, mechanical, and shear forces under controlled moisture conditions, promoting protein denaturation, molecular alignment, aggregation, and cross-linking through disulfide bonds, hydrogen bonding, hydrophobic interactions, ionic interactions, and van der Waals forces. These molecular transformations lead to the formation of structured protein networks with mechanical and sensory characteristics similar to those of conventional meat products [3,4].

To date, producing meat analogues has relied primarily on soy protein, wheat gluten, pea protein, and other legume-derived protein ingredients. Among them, soy protein isolate remains one of the most extensively used raw materials because of its excellent gelation capacity, water-binding properties, and extrusion performance. However, the production of protein isolates requires intensive extraction and purification processes, resulting in increased processing costs, the generation of side streams, and the partial loss of naturally occurring bioactive compounds, dietary fiber, and micronutrients [5,6]. Consequently, the use of whole grain flour has emerged as a more sustainable alternative aligned with circular economy principles, as these ingredients preserve the entire nutritional matrix while requiring substantially less industrial processing.

Despite their sustainability advantages, whole flours generally contain lower protein concentrations than purified protein ingredients, which may limit their direct application in highly structured meat analogue systems. In this regard, germination has gained considerable attention as a natural and cost-effective bioprocessing strategy capable of enhancing both the nutritional and functional properties of legumes. Germination activates endogenous enzymes that promote biochemical modifications, including partial protein hydrolysis, the degradation of antinutritional factors, increased phenolic compound content, improved antioxidant activity, and enhanced protein functionality. In particular, endogenous proteases and amylases partially hydrolyze storage proteins and starch, reduce matrix compactness, and expose hydrophilic and hydrophobic groups, thereby modifying water solubility and oil-binding behavior [7]. Recent studies have demonstrated that germinated Andean lupin exhibits improved techno-functional characteristics and greater potential for application in plant-based food formulations compared with its non-germinated counterpart [8].

Germination and extrusion therefore induce complementary biochemical and structural transformations in plant-based matrices. Following the enzymatic modifications generated during germination, extrusion promotes starch gelatinization, disruption of crystalline structures, protein unfolding, molecular rearrangement, and aggregation. Controlled protein denaturation may enhance protein interactions with water and oil, whereas excessive aggregation may decrease solubility and restrict the accessibility of digestive enzymes [9]. These transformations are not governed exclusively by temperature and shear but are also influenced by pH, protein surface charge, and interactions with non-protein compounds. Changes in the ionization state of proteins regulate electrostatic attraction and repulsion, thereby affecting molecular assembly, hydration, and aggregation. Evidence from pH-driven encapsulation systems indicates that structural and charge variations can modify the protein–ligand association and macromolecular organization [10]. Similarly, metal–phenolic networks can reorganize protein interfaces and alter the formation and stability of interfacial structures [11]. Although these studies were conducted using model colloidal systems, their findings provide a mechanistic framework for understanding how phenolic compounds, protein–starch interactions, and charge-dependent protein–non-protein associations may influence the water solubility index (WSI), oil absorption capacity (OAC), cooking loss, texture, and protein digestibility of germinated and extruded food matrices.

The successful development of fibrous meat analogues depends on complex interactions among protein composition, hydration behavior, rheological properties, and extrusion processing conditions. The charge-regulated assembly of proteins and their interactions with starch and phenolic compounds may additionally modulate the hydration, aggregation, and stabilization of the resulting protein network. These factors directly influence key technological attributes such as texture, cohesiveness, elasticity, water-holding capacity, and overall structural integrity, which are critical determinants of consumer acceptance and product quality [12]. Although the application of lupin-derived ingredients in meat analogues has increased in recent years, limited information is available regarding the use of germinated Andean lupin flour as a partial replacement for soy protein isolate in high-moisture extrusion systems. Furthermore, the effects of such substitution on technological performance, protein quality, and functional characteristics of extruded meat analogues remain largely unexplored.

To address these knowledge gaps, this study investigated the feasibility of utilizing germinated Andean lupin whole flour as a sustainable, partial substitute for soy protein isolate in high-moisture extruded meat analogue systems. Response surface methodology combined with chemometric approaches was employed to optimize formulation and processing conditions. The optimized meat analogue was subsequently characterized in terms of its techno-functional properties, instrumental color, texture, amino acid composition, in vitro protein digestibility, and nutritional profile, providing a comprehensive assessment of its potential as a high-quality plant-based protein product.

## 2. Materials and Methods

### 2.1. Materials

Andean lupin seeds (*Lupinus mutabilis* Sweet var. Andenes; 2023 harvest) were provided by the *Programa de Cereales y Granos Nativos* of the *Universidad Nacional Agraria La Molina* (UNALM, Lima, Peru). The seeds underwent pilot-scale germination in a BJPX-HT400II germination chamber (Maquilak, Jinan, China) under optimized parameters (22.6 °C, 96 h, and 80% relative humidity) as specified by Paucar-Menacho et al. [2]. The resulting sprouts were dried in a semi-industrial SBT-10XL tray dryer (Thorr, Junín, Peru) at 45 °C for 30 h under forced-air circulation and subsequently pulverized using an MDMT-60XL hammer mill (Thorr, Junín, Peru) with a 0.5 mm sieve, yielding germinated Andean lupin whole flour. This flour was vacuum-packaged in high-density polyethylene bags and stored at −18 °C in an RCFB32 freezer (Midea, Rio Grande do Sul, Brazil).

Commercial soy protein isolate (SSPI-90G, Quimtia S.A., Lima, Peru) was utilized as the primary protein matrix. The chemicals and reagents used in this study included sodium hypochlorite, sulfuric acid, boric acid, copper sulfate, potassium sulfate, sodium sulfate, sodium hydroxide, hydrochloric acid, monosodium phosphate monohydrate, disodium phosphate heptahydrate, and trichloroacetic acid, all purchased from Synth (Diadema, Brazil). The amino acid standards (LAA21-1KT), pepsin from porcine gastric mucosa (≥400 U·mg^−1^ protein), trypsin from porcine pancreas (≥250 U·mg^−1^ protein), toluene, phenol, methanol, cloroform, sodium borate, diethyl ethoxymethylenemalonate (DEEMM), sodium acetate, and acetonitrile were obtained from Sigma-Aldrich (St. Louis, MO, USA).

### 2.2. Experimental Design

To systematically evaluate formulation and processing effects, a 2^2^ central composite design was adopted for meat analogue development. The independent variables included the germinated Andean lupin whole flour-to-soy protein isolate ratio (X_1_) and feed moisture conditioning (X_2_). Their experimental boundaries were established through preliminary extrusion runs to ensure stable processability and satisfactory macroscopic structuring. Coded and actual levels of the experimental design are detailed in Table 1.

The experimental design included three replicated center points (Trials 9–11), as recommended for Central Composite Designs, to obtain an unbiased estimate of the pure experimental error and to evaluate the lack-of-fit of the proposed regression models statistically. Furthermore, the replicated center points provided information on process repeatability and experimental variability, increasing the reliability of the fitted response surface models and numerical optimization.

### 2.3. Obtaining the High-Moisture Extruded Meat Analogues

The high-moisture extrusion process was conducted in a TwinLab-F 20/40 co-rotating intermeshing twin-screw extruder (Brabender GmbH, Duisburg, Germany; screw diameter: 20 mm; L/D ratio = 40). The screw geometry integrated five conveying elements (SE/30/30), four conveying elements (SE/20/20), two SE/30/30 elements, one kneading block (KP45/5/20), and alternating SE/30/30 and SE/20/20 elements up to the die zone to optimize mixing, shear intensity, and flow homogeneity.

**Figure 1:**
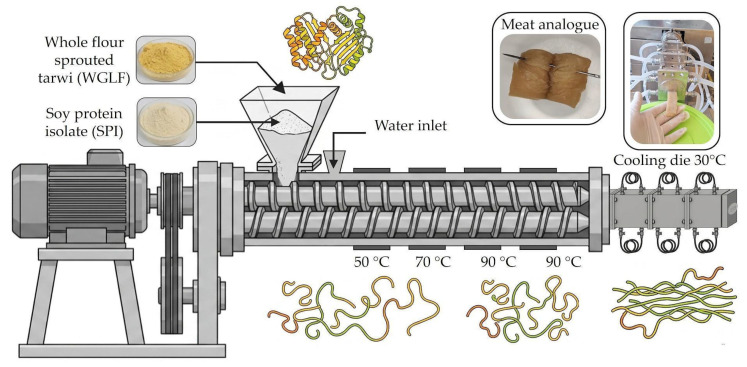
Schematic representation of the high-moisture extrusion process used to produce plant-based meat analogues formulated with germinated Andean lupin whole flour and soy protein isolate.

The throughput was fixed at 10 kg·h^−1^ and the screw speed at 300 rpm. Processing temperatures across the four-barrel zones were regulated at 50, 70, 90, and 90 °C, respectively. To promote directional structuring and stabilize the extruded matrix, a rectangular cooling die (450 mm in length, 7.5 mm in height, and 33.5 mm in width) was coupled to the extruder and maintained at 30 °C. The complete extrusion setup is schematically depicted in Figure 1.

Feed moisture conditioning and the germinated Andean lupin whole flour-to-soy protein isolate ratio were regulated based on the experimental matrix detailed in Table 1. Once extruded, the samples were stabilized at room temperature, sealed in high-density polyethylene bags, and refrigerated in an industrial refrigerator (BF Cozinhas, São Paulo, Brazil) at 4 °C until subsequent physicochemical, technological, nutritional, and functional evaluations.

### 2.4. Proximate Composition of Germinated Andean Lupin Whole Flour and Soy Protein Isolate

Proximate profiles of the germinated Andean lupin whole flour and soy protein isolate were determined in accordance with the Association of Official Analytical Chemists methods AOAC [13]: moisture (method 934.01), ash (method 942.05), crude protein (Kjeldahl method 920.152, N × 6.25), and crude lipid (method 960.39). Total carbohydrates were calculated by difference. All measurements were performed in triplicate.

### 2.5. Technofunctional Properties

#### 2.5.1. Water Absorption Index (WAI) and Water Solubility Index (WSI)

The WAI and WSI were determined according to the method of Anderson et al. [14] with modifications by Lima et al. [15]. Pre-dried extrudates (80 °C for 18 h in an Elas-50 Lts convection oven, Ovens Medic, Lima, Peru) were milled (AS 200 knife mill, Fritsch, Idar-Oberstein, Germany) to a particle size of <80 µm before testing.

A 1.0 g aliquot (in triplicate) was hydrated in 10 mL of distilled water for 15 min and centrifuged (Model 0408-1, Kert Lab, Lima, Peru) at 2000 × *g* for 25 min. The supernatant was dried at 105 °C to a constant mass to quantify soluble matter, while the sediment was weighed to assess the water-retained fraction. Calculations for the WAI and WSI followed Equations (1) and (2), respectively.

(1) $$WAI=\frac{M_{rc}}{\left({M_{a}-M}_{re}\right)}$$

(2) $$WSI=\frac{M_{re}}{M_{a}}\times 100$$

where $M_{rc}$ corresponds to the mass of the centrifugation residue (g), $M_{a}$ to the initial dry mass of the sample (g), and $M_{re}$ to the mass of the residue obtained after evaporation of the supernatant (g).

#### 2.5.2. Oil Absorption Capacity (OAC)

OAC was determined according to the method proposed by Wang et al. [16] with minor modifications, in triplicate. Dried and milled extrudates with a homogeneous particle size were used for the evaluation. A 1.0 g aliquot (dry basis) was mixed with 10 mL of commercial soybean oil in a pre-weighed centrifuge tube, allowed to stand at room temperature for 15 min, and centrifuged at 2000 × *g* for 25 min. The supernatant was quantitatively removed, and the remaining oil-retaining residue was weighed. Calculations for OAC followed Equation (3).

(3) $$OAC=\frac{M_{rc}}{M_{a}}\times 100$$

where $M_{rc}$ corresponds to the mass of the centrifugation residue (g) and $M_{a}$ to the initial dry mass of the sample (g).

#### 2.5.3. Cooking Rehydration Capacity (CRC) and Cooking Loss (CL)

CRC and CL were determined based on the method described by Zhou et al. [17], with modifications. The extruded samples were refrigerated (4 °C). A 10.0 g sample was cooked in boiling distilled water (1:10 *w*/*v* ratio) for 4 min, in triplicate. The cooked samples were then drained on a sieve for 2 min to remove surface moisture and immediately weighed.

The residual cooking water was transferred to pre-weighed Petri dishes, evaporated at 85 °C for 18 h, and dried at 105 °C for 2 h to a constant mass to determine the leached solids. Calculations for CRC and CL followed Equations (4) and (5), respectively. CRC was defined as the percentage of water absorbed during cooking, whereas CL represented the percentage of solids lost to the medium based on the initial sample mass.

(4) $$CRC\;(\%)=\frac{\left(M_{ac}-M_{ia(bs)}\right)}{\left(M_{ia\left(bs\right)}-M_{sl}\right)}\times 100$$

(5) $$CL\;(\%)=\frac{M_{sl}}{M_{ia(bs)}}\times 100$$

where $M_{ac}$ corresponds to the mass of the sample after cooking (g), $M_{ia(bs)}$ to the initial dry mass of the sample (g), and $M_{sl}$ to the mass of solids leached during cooking (g).

#### 2.5.4. Instrumental Color

Extrudate surface color was assessed using a CR-310 colorimeter (Konica Minolta, Osaka, Japan) under the CIELAB system (L*: lightness; a*: redness/greenness; b*: yellowness/blueness) [18]. Multiple surface readings were averaged from three independent samples to ensure statistical representativeness.

### 2.6. Textural Profile Analysis (TPA)

Texture attributes were measured using an Instron Model 3365 universal testing machine (Instron Corp., Norwood, MA, USA) with a 500 N load cell. TPA was performed using an S21889 compression probe(Instron Corp., Norwood, MA, USA) (42 mm diameter, 8 mm height), while shear force (SF) was recorded using a Warner–Bratzler blade (S21890) (Instron Corp., Norwood, MA, USA). The analysis was performed with six repetitions.

Uniformly cut samples (20 mm × 20 mm edge and 7 mm height) were tested at a crosshead speed of 1 mm·s^−1^. Hardness, cohesiveness, springiness, chewiness, gumminess, and adhesiveness were determined from the TPA curves. The peak cutting force during shearing was recorded as SF.

### 2.7. Numerical Optimization and Validation of the Mathematical Model

Quadratic regression models were fitted to the technological data using Design-Expert version 13 (Stat-Ease Inc., Minneapolis, MN, USA). Response Surface Methodology was applied to establish predictive equations and map the experimental space via 3D response surface plots.

Multi-response numerical optimization was executed via the desirability function method [19]. Optimization goals (maximization, minimization, or target value) were assigned to significant responses based on desired meat analogue characteristics, and their relative importance was rated on a scale from 1 to 5. Only models meeting significance (*p* < 0.10) and high goodness-of-fit thresholds (R^2^ > 0.75) were considered.

The optimum formulation and operating parameters were established by maximizing the global desirability function (D) within the experimental boundaries. To validate the accuracy of the mathematical models, the optimized product was manufactured under the predicted optimal conditions and evaluated in triplicate. Finally, the observed experimental values were statistically compared against the model-predicted intervals.

### 2.8. Amino Acid Profile

The amino acid composition was determined following acid hydrolysis of the protein fraction and subsequent high-performance liquid chromatography (HPLC) analysis. Briefly, samples containing approximately 20 mg of protein (dry basis) were transferred to vacuum hydrolysis tubes and 5 mL of 6 mol·L^−1^ hydrochloric acid containing 0.1% phenol (*w*/*v*) was added. The tubes were vacuum-sealed and incubated at 110 °C for 24 h. After hydrolysis, the hydrolysates were neutralized with 6 mol·L^−1^ NaOH to pH 7.0, filtered, and adjusted to a final volume of 25 mL.

Protein hydrolysates were derivatized with diethyl ethoxymethylenemalonate (DEEMM) according to Gómez-Alonso et al. [20]. For derivatization, 1.75 mL of 1 M borate buffer (pH 9.0), 750 μL of methanol, 1 mL of sample, and 30 μL of DEEMM were mixed, sonicated for 30 min using a CBU/100/3LDG ultrasonic bath (Planatec, São Paulo, Brazil) at 40 kHz (100 W), and subsequently heated at 70 °C for 2 h in a static SL-150 water bath (Solab, Piracicaba, Brazil).

The derivatized samples were analyzed using an HPLC system (Agilent 1260 Infinity, Agilent Technologies, Santa Clara, CA, USA) equipped with a G1314B diode-array detector (Agilent Technologies, Santa Clara, CA, USA). Chromatographic separation was performed on a Zorbax Eclipse Plus C18 column (4.6 × 250 mm, 5 μm) maintained at 16 °C. A binary gradient elution system was employed using mobile phase A consisting of 50 mM acetate buffer (pH 8.75) and mobile phase B consisting of acetonitrile–water (80:20, *v*/*v*). The flow rate was set at 0.9 mL min^−1^.

The gradient program was as follows: 0–20 min, 90% A and 10% B; 30.5–33.5 min, 83% A and 17% B; 65 min, 60% A and 40% B; 73 min, 28% A and 72% B; 78 min, 18% A and 82% B; and 82–85 min, 100% B. Detection was performed at 280 nm. Amino acids were identified by comparing retention times with those of authentic standards and quantified using external calibration curves prepared for each amino acid. The amino acid composition was expressed as g·100 g^−1^ protein (d.b.) and subsequently used to evaluate changes in protein quality associated with the partial replacement of soy protein isolate by germinated Andean lupin whole flour.

### 2.9. In Vitro Protein Digestibility

In vitro protein digestibility was determined using a simulated gastrointestinal digestion procedure according to the method described by Pan et al. [21], involving sequential enzymatic hydrolysis with pepsin and trypsin. The nitrogen content of the samples before and after digestion was determined according to American Association of Cereal Chemits International Method 46-13.01 [22]. In vitro protein digestibility values were calculated using Equation (6) and expressed as the percentage of protein digested during the simulated gastrointestinal process.

(6) $$IVPD\;\left(\%\right)=\frac{\left(N_{t}-N_{b}-N_{e}\right)}{\left(N_{t}\right)}\times 100$$

where *IVPD* corresponds to in vitro protein digestibility (%), $N_{t}$ to the total nitrogen content, $N_{b}$ to the nitrogen content of the blank, and $N_{e}$ to the nitrogen content after simulated in vitro enzymatic digestion.

### 2.10. Statistical Analysis

All measurements were performed in triplicate, except for texture profile analysis, which was conducted with six replicates. Data normality was assessed using the Shapiro–Wilk test (*p* < 0.05). A significance level of *p* < 0.10 (90% confidence) was adopted for Response Surface Methodology because the primary objective of this study was process optimization rather than confirmatory hypothesis testing, using a Statistica 7.0 software (StatSoft Inc., Tulsa, OK, USA). In optimization studies employing central composite designs, a 90% confidence level is frequently used to reduce the probability of excluding potentially relevant factors during model development. Nevertheless, statistical significance alone was not considered sufficient for model selection. Only models exhibiting significant regression (*p* < 0.10), satisfactory goodness-of-fit (R^2^ > 0.75), non-significant lack-of-fit, and successful experimental validation were considered suitable for prediction and optimization. Responses that did not meet these criteria were interpreted descriptively and excluded from the optimization procedure. Before multivariate analysis, the data were log_10_-transformed and Pareto-scaled. Multivariate analyses, including principal component analysis (PCA) and partial least squares discriminant analysis (PLS-DA), were performed on the amino acid profile and in vitro protein digestibility data to evaluate sample discrimination and overall variability using MetaboAnalyst 6.0 (Xia Lab, Montreal, QC, Canada).

## 3. Results

### 3.1. Proximate Composition of Raw Materials

The proximate composition of the germinated Andean lupin whole flour used in this study was previously determined and reported by our research group as part of a germination optimization study [2]. Under the optimized germination conditions, the flour contained 45.36% protein, 17.76% lipids, 5.27% ash, 23.82% total dietary fiber, and 9.68% available carbohydrates on a dry basis. These results highlight the nutritional value of germinated Andean lupin whole flour as a protein-rich ingredient with substantial amounts of dietary fiber and bioactive compounds, making it a promising raw material for plant-based meat analogue formulations.

The high protein content observed is consistent with the intrinsic composition of germinated Andean lupin, which is one of the richest protein sources among grain legumes. In addition, germination promotes biochemical modifications associated with reserve mobilization, enzymatic activity, and the degradation of antinutritional factors, thereby improving the nutritional quality and functional performance of the resulting flour. Previous studies have demonstrated that optimized germination conditions enhance the nutritional profile and techno-functional properties of Andean lupin, supporting its application in structured plant-based foods and extrusion-based products [2].

The soy protein isolate used in this study contained 88.16 ± 0.61% protein, 0.17 ± 0.01% lipids, 4.60 ± 0.11% ash, and 7.07 ± 0.02% total carbohydrates. This protein content falls within the range commonly reported for commercial soy protein isolates, reflecting the high degree of purification achieved during protein isolation. The low lipid and carbohydrate contents further confirm the suitability of soy protein isolate as a structuring ingredient for high-moisture extrusion processes, where concentrated protein sources are required to promote the formation of fibrous structures.

Overall, the compositional characteristics of both raw materials highlight their complementary nutritional and technological roles in the formulation of plant-based meat analogues. Germinated Andean lupin whole flour contributes high-quality protein, dietary fiber, lipids, and micronutrients. In contrast, soy protein isolate provides a highly concentrated protein fraction source that promotes the development of cohesive fibrous structures during high-moisture extrusion. Consequently, their combination may enable the production of nutritionally improved meat analogues while partially reducing the dependence on highly refined protein ingredients. Furthermore, the use of germinated Andean lupin whole flour aligns with current trends toward minimally processed ingredients and more sustainable plant-based food systems.

### 3.2. Effect of Formulation on the Techno-Functional Properties of Extruded Meat Analogues

Table 2 presents the techno-functional profiles of the texturized meat analogues obtained across the different experimental trials. Evaluated responses encompassed hydration behavior (WAI, WSI, OAC, CRC, and CL), surface color, and instrumental texture attributes. The distinct variations observed across the experimental matrix confirm that the germinated Andean lupin whole flour-to-soy protein isolate ratio and feed moisture content are critical determinants of the structural and functional characteristics of the extruded matrices.

#### 3.2.1. Water Absorption Index and Water Solubility Index

The WAI values ranged from 3.72 to 5.62% across the evaluated formulations (Table 2). Statistical analysis indicated that neither the germinated Andean lupin whole flour-to-soy protein isolate ratio (X_1_) nor the feed moisture conditioning (X_2_) exerted significant effects on this technological attribute (*p* > 0.10). Furthermore, the fitted regression model exhibited a low coefficient of determination (R^2^ = 0.65), indicating insufficient predictive capacity for response surface methodology. These findings imply that the thermomechanical conditions applied during extrusion promoted the development of macromolecular networks with uniform hydration capacities, irrespective of formulation or moisture variations. Consequently, WAI was excluded as an optimization criterion.

Conversely, the WSI was highly sensitive to the experimental variables, ranging from 6.21 to 18.79% (Table 2). The quadratic polynomial model fitted to this response exhibited excellent predictive performance (R^2^ = 0.96), indicating that variations in the WSI were strongly influenced by both the GAL ratio and feed moisture content. Analysis of variance (ANOVA) confirmed the significance of the linear and quadratic effects of both independent variables (*p* < 0.10), indicating that formulation composition and processing moisture significantly influenced the water solubility of the extruded matrix.

The positive regression coefficient obtained for the germinated Andean lupin whole flour fraction (X_1_) indicated that higher inclusion levels favored greater material solubilization. This phenomenon is likely driven by the accumulation of low-molecular-weight peptides, free sugars, and partially hydrolyzed polymers generated during seed germination. This biological bioprocessing activates endogenous hydrolases that modify the architectural arrangement of seed storage reserves, rendering them more prone to thermal degradation and solubilization under high-shear extrusion.

**Figure 2:**
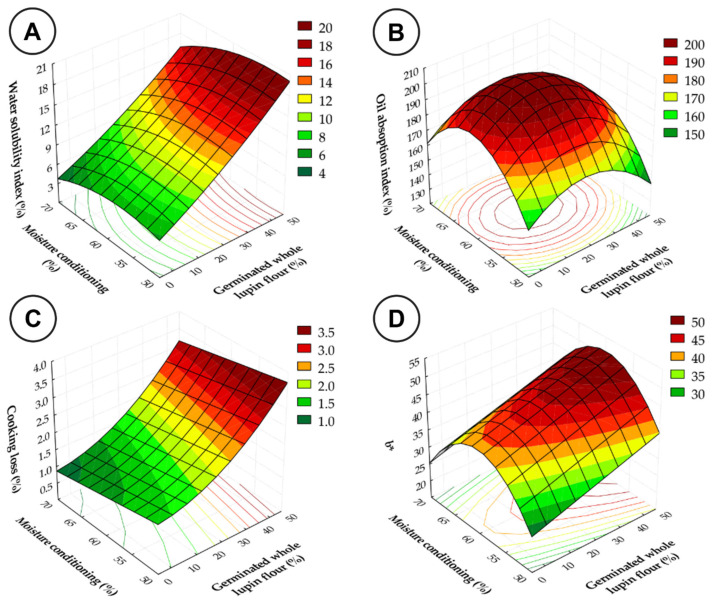
Contour plots generated from the response surface models showing the effects of the germinated Andean lupin whole flour–soy protein isolate ratio (X_1_) and feed moisture content (X_2_) on selected techno-functional properties of high-moisture extruded meat analogues: (**A**) water solubility index (WSI); (**B**) oil absorption capacity (OAC); (**C**) cooking loss (CL); and (**D**) CIELab yellowness parameter (b*).

On the other hand, feed moisture content (X_2_) exerted a pronounced negative impact on the WSI, indicating that elevated hydration levels restricted solute leaching from the texturized matrix. In high-moisture extrusion systems, water acts as a plasticizer, reducing melt viscosity and shear stress while facilitating protein unfolding, molecular mobility, aggregation, and alignment, ultimately promoting the formation of a continuous fibrous protein network. This structural rearrangement may result in a more cohesive and directionally organized matrix that resists aqueous breakdown. The final predictive equation is detailed in Equation (7), and the corresponding two-dimensional contour plot is displayed in Figure 2A. The solubility increments induced by germinated whole flours are consistent with the established literature on malted or germinated pulses, where macromolecular cleavage disrupts protein–carbohydrate matrix interactions during mechanical texturization.

(7) $$\mathrm{WSI}\;(\%)=12.12+4.72x_{1}+0.41{x_{1}}^{2}-1.06x_{2}-0.79{x_{2}}^{2}-0.27x_{1}x_{2}$$

where WSI represents the water solubility index, while x_1_ and x_2_ correspond to the coded values of the germinated Andean lupin whole flour–soy protein isolate ratio and feed moisture content, respectively.

The structural behavior of the WSI stems from complex macromolecular modifications induced by high-moisture extrusion within the protein-rich matrix. During thermal texturization, the concomitant action of thermal energy, mechanical shear, pressure, and hydration drives protein denaturation, subunit unfolding, subsequent aggregation, and supramolecular reorganization, altering the physicochemical properties of the extrudates. These severe thermomechanical transformations facilitate the cleavage and liberation of low-molecular-weight fragments and soluble fractions, thereby enhancing water solubility.

Here, the increased WSI resulting from germinated Andean lupin whole flour inclusion is linked to the accumulation of pre-existing soluble solutes (short-chain peptides, free amino acids, and oligosaccharides) during germination. This biological pretreatment triggers endogenous hydrolases that break down storage reserves into low-molecular-weight, highly soluble constituents. Because these constituents partially resist thermal texturization or bypass macromolecular cross-linking during extrusion, the resulting lupin-dense formulations exhibit an enhanced propensity to leach solutes upon hydration.

This mechanism aligns with the established literature on plant-protein extrusion. For instance, Meng et al. [23] demonstrated that texturized soybean proteins undergo pronounced swelling, denaturation, and structural rearrangement, fundamentally altering matrix solubility. Similarly, Cho and Ryu [24] reported that although soy protein isolates exhibit high intrinsic nitrogen solubility, their hydration and solubility properties are strongly influenced by processing conditions and formulation composition. The strong influence of feed moisture underscores water’s role as a critical plasticizer within the extruder barrel. Elevated moisture attenuates specific mechanical energy input and lowers melt viscosity, reducing shear-induced fragmentation and promoting well-integrated, cohesive protein networks that restrict solute leaching.

Interestingly, the lack of significant variance in WAI contrasted with the high sensitivity of the WSI indicates that the overall water-holding capacity of the macromolecular network remained uniform across the experimental domain, irrespective of local matrix solubilization. This suggests that the germinated Andean lupin whole flour-to-soy protein isolate ratio and feed moisture conditioning primarily modulated the intensity of molecular degradation rather than the total hydrophilic binding sites of the texturized network. Consequently, the WSI serves as a far more sensitive probe for tracking structural modifications in high-moisture extruded meat analogues. Furthermore, the high dietary fiber content of the germinated lupin whole flour likely reinforced these solubility trends, as germination is known to shift insoluble-to-soluble fiber balances and alter downstream protein–carbohydrate interactions within the extruded shear plane.

#### 3.2.2. Oil Absorption Capacity

The OAC values varied between 166.57 and 239.42% across the experimental formulations (Table 2). The fitted quadratic model displayed a high coefficient of determination (R^2^ = 0.93), confirming that the independent variables satisfactorily accounted for the variation in OAC. Analysis of variance (ANOVA) revealed significant linear and quadratic effects (*p* < 0.10) for the germinated Andean lupin whole flour-to-soy protein isolate blending ratio (X_1_) and feed moisture content (X_2_).

The regression coefficients revealed that higher inclusion levels of germinated Andean lupin whole flour negatively impacted OAC, implying a diminished capacity of the texturized matrix to entrap lipids. This trend is likely associated with the diluted protein concentration and higher dietary fiber content of the whole lupin flour relative to commercial soy protein isolate. Given that oil immobilization is fundamentally governed by the physical entrapment of oil and the binding of lipids to exposed hydrophobic domains, substituting highly purified soy protein isolate with whole pulse flour restricts the density of accessible nonpolar binding sites.

Concurrently, feed moisture conditioning exerted a positive linear but negative quadratic effect on OAC, revealing that intermediate moisture levels maximized oil retention, whereas excessive hydration hindered it. This performance reflects the dual role of water during thermomechanical processing, while moderate moisture facilitates macromolecular plasticization, protein unfolding, and the exposure of buried hydrophobic residues, excess moisture attenuates specific mechanical energy (SME) input and barrel shear stress. This reduction in shear minimizes structural breakdown, driving the formation of highly integrated, compact protein networks with fewer exposed nonpolar interaction sites.

These findings highlight the combined influence of raw material composition and processing conditions on the lipid-binding properties of texturized meat analogues. Since oil retention critically dictates flavor stabilization, mouthfeel, and juiciness, tailoring this technological parameter is vital for optimizing the palatability of plant-based matrices. Additionally, the baseline lipid fraction of the germinated lupin flour may have accelerated this OAC decline, as endogenous lipids can saturate internal hydrophobic pockets within the matrix, hindering the retention of external oil during testing. The final predictive equation for OAC is presented in Equation (8), and its response surface contour is depicted in Figure 2B.

(8) $$\mathrm{OAC}\;(\%)=204.56-3.36x_{1}-10.66{x_{1}}^{2}+3.64x_{2}-16.06{x_{2}}^{2}$$

where OAC represents the oil absorption capacity, while x_1_ and x_2_ correspond to the coded values of the germinated Andean lupin whole flour–soy protein isolate ratio and feed moisture content, respectively.

The downward trend in OAC associated with increasing germinated Andean lupin whole flour levels can be ascribed to divergent functional properties between lupin and soy proteins, coupled with structural transitions within the texturized matrix. Piornos et al. [25] highlighted that lupin storage proteins possess inherently lower lipid-binding capacities than those of soy, offering a rationale for the attenuated oil retention in lupin-dense formulations. Given that lipid immobilization relies extensively on the spatial distribution of hydrophobic amino acid residues and the structural architecture of the matrix, replacing the purified SPI with whole flour dilutes the density of accessible nonpolar anchoring sites.

Beyond intrinsic protein functionality, the macro-component profile of germinated Andean lupin whole flour plays a pivotal role in these outcomes. Unlike ultra-purified SPI, this whole pulse flour introduces a complex matrix rich in dietary fiber and native lipids, unavoidably modulating macromolecular protein–protein and protein–lipid cross-linking during thermal extrusion. The incorporation of these non-protein constituents acts as a physical barrier, disrupting the continuity of the proteinaceous network, thereby decreasing the oil-retention capacity of the extruded matrix. Concurrently, native lipids pre-existing in the germinated seed matrix may pre-occupy hydrophobic binding pockets, reducing the net availability for exogenous lipid adsorption during analysis.

This structural disruption aligns with past research addressing pulse inclusion in texturized systems. For instance, Elhordoy et al. [26] highlighted that elevated lupin incorporation yielded softer and less cohesive textures in high-moisture meat analogues, indicating compromised network integrity and a less dense macromolecular assembly. Such poorly integrated protein networks likely lack the capillary forces and interaction sites necessary for efficient lipid fixation, exacerbating the OAC decline.

Systematically, these insights confirm that OAC is highly sensitive to the interplay between formulation composition and hydration variables. While an SPI-dominant framework optimizes oil immobilization, the progressive substitution with germinated Andean lupin whole flour creates a more open, lipid-saturated structure that curtails this property. Technologically, managing this balance is paramount, as oil retention directly governs flavor stabilization, mouthfeel, and juiciness in meat alternatives. Consequently, tailoring the lupin flour-to-soy protein isolate blending ratio emerges as a crucial strategy to balance the unique nutritional advantage of lupin’s native lipid profile with the strict structural demands of high-moisture extrudates.

#### 3.2.3. Cooking Rehydration Capacity and Cooking Loss

The CRC of the extruded meat analogues ranged from 8.46 to 81.53% across the evaluated formulations (Table 2). Although some individual regression terms exhibited marginal significance (*p* < 0.10), the overall regression model showed low explanatory power (R^2^ = 0.59) and was therefore considered inadequate for predictive modeling. Consequently, CRC was not included in the numerical optimization, and its behavior was discussed only descriptively.

The lack of significant effects suggests that the rehydration behavior of the extruded products was not primarily governed by the formulation variables investigated in this study. Instead, CRC may have been more strongly influenced by microstructural characteristics developed during high-moisture extrusion, including pore size distribution, fiber alignment, protein network density, and the extent of molecular interactions established during cooling and structuring.

In contrast, CL ranged from 1.01 to 3.84% (Table 2) and was satisfactorily described by the fitted quadratic model (R^2^ = 0.91). Analysis of variance revealed significant linear and quadratic effects of the germinated Andean lupin whole flour–soy protein isolate ratio (*p* < 0.05), whereas feed moisture content exerted a significant influence only through its linear term. Neither the quadratic effect of moisture content nor the interaction between the independent variables was statistically significant.

The regression coefficients indicated that increasing the proportion of germinated Andean lupin whole flour resulted in higher cooking loss values. This behavior may be attributed to its lower protein concentration and higher contents of dietary fiber, endogenous lipids, and other non-protein constituents compared with soy protein isolate. The lower proportion of soy protein isolate may have limited the formation of a continuous and cohesive protein network, thereby favoring the leaching of soluble components during cooking.

The fitted regression model describing cooking loss is presented in Equation (9), and the corresponding contour plot is shown in Figure 2C.

(9) $$\mathrm{CL}\;(\%)=1.87+0.81x_{1}+0.22{x_{1}}^{2}-0.21x_{2}$$

where CL represents cooking loss, while x_1_ and x_2_ correspond to the coded values of the germinated Andean lupin whole flour–soy protein isolate ratio and feed moisture content, respectively.

In addition to dietary fiber and endogenous lipids, phenolic compounds and other low-molecular-weight constituents released during germination, including small peptides, free amino acids, organic acids, and soluble carbohydrates, may also have influenced matrix organization. Germination-induced hydrolysis reduces matrix compactness and increases the accessibility of hydrophilic and hydrophobic groups, whereas extrusion promotes protein unfolding, starch gelatinization, molecular rearrangement, and the formation of new protein–protein and starch–protein interactions. Functional groups exposed during protein denaturation can interact with starch through hydrogen bonding and other intermolecular associations, thereby modifying the three-dimensional stability, solubility, hydration, and oil-retention behavior of the matrix [27]. Phenolic compounds may also associate with exposed protein regions through hydrogen bonding, hydrophobic interactions, and electrostatic forces. Depending on their concentration and affinity for protein surfaces, these associations may promote intermolecular bridging and interfacial stabilization or competitively interfere with protein–protein interactions, thereby reducing the continuity of the fibrous network [10,11].

This combined mechanism may help explain the increase in the WSI and cooking loss and the reduction in OAC observed as the proportion of germinated Andean lupin whole flour increased. The greater abundance of soluble peptides, phenolics, carbohydrates, and fiber fragments may increase the amount of material released into the aqueous phase, whereas protein–phenolic and protein–lipid associations may mask hydrophobic regions or reduce their accessibility for oil retention. Téllez-Morales et al. [27] reported that extrusion modified the WSI and reduced OAC in starch–protein systems through protein denaturation, starch–protein interactions, and changes in the availability of nonpolar residues. Similarly, Singh et al. [28] observed changes in starch degradation, molecular interactions, solubility, hardness, and microstructure in extruded cereal–legume systems. In addition, lipid oxidation products generated during extrusion may promote protein aggregation and cross-linking, thereby altering matrix porosity, hydration behavior, and oil retention [9].

Previous studies have reported similar relationships between formulation composition and cooking stability in plant-based meat analogues. Oukhmanou-Bensidhoum et al. [29] observed moderate cooking losses in plant-based burgers formulated with lentil, pea, and black bean ingredients, attributing this behavior to the maintenance of adequate structural integrity during heating. Similarly, Peñaranda et al. [30] demonstrated that extrusion-induced texturization of pea protein formulations enhanced matrix stability and reduced the release of soluble compounds during cooking. Collectively, these findings support the hypothesis that the formation of a continuous and well-organized protein network is a major determinant of cooking stability in plant-based meat systems.

Feed moisture content showed a negative effect on cooking loss, indicating that higher moisture levels improved matrix stability and reduced solid release. During high-moisture extrusion, water acts as a plasticizer that facilitates molecular mobility, partial protein unfolding, rearrangement, and intermolecular interactions. These conditions may favor the development of a more cohesive matrix capable of retaining soluble components and resisting structural disruption during cooking.

Overall, the low cooking loss values obtained across all formulations indicate adequate structural integrity of the extruded products. Whereas CRC could not be satisfactorily explained by the evaluated variables, cooking loss depended on both formulation composition and feed moisture content. Increasing the proportion of germinated Andean lupin whole flour favored the release of soluble material, while higher feed moisture levels improved matrix stability and reduced cooking losses.

#### 3.2.4. Instrumental Color Parameters

Color is an important quality attribute of plant-based meat analogues because it directly influences consumer perception and product acceptability. The instrumental color of the extruded meat analogues was evaluated using the CIELab color system, where L* represents lightness, a* the red–green coordinate, and b* the yellow–blue coordinate. The results obtained for the different formulations are presented in Table 3.

The lightness parameter (L*), which ranges from black (0) to white (100), varied from 65.95 to 84.79 across the evaluated formulations (Table 3). Analysis of variance revealed that neither the germinated Andean lupin whole flour–soy protein isolate ratio nor feed moisture content significantly affected lightness (*p* = 0.558). Furthermore, the fitted model exhibited a low coefficient of determination (R^2^ = 0.43), indicating limited predictive performance. These findings suggest that the formulation and processing variables investigated were insufficient to explain the observed variability in product lightness and that additional factors associated with extrusion-induced structural and physicochemical changes may have contributed to the color differences observed among formulations.

Similarly, the redness parameter (a*), which describes color variation along the green (−) to red (+) axis, ranging from 3.62 to 8.05. No significant effects of the evaluated factors were detected for this response (*p* = 0.572), and the fitted model exhibited poor predictive ability (R^2^ = 0.25). Therefore, the formulation variables investigated in the present study did not substantially influence the development of red color tones in the extruded meat analogues.

In contrast, the yellowness parameter (b*), corresponding to the blue (−) to yellow (+) axis, varied markedly among formulations, ranging from 26.33 to 50.17. Regression analysis revealed a significant effect of the germinated Andean lupin whole flour–soy protein isolate ratio (*p* = 0.010), while the quadratic term of feed moisture content also significantly influenced this response. The fitted model demonstrated satisfactory predictive performance (R^2^ = 0.81), indicating that the experimental variables adequately explained the observed variation in b* values.

The positive effect of germinated Andean lupin whole flour on yellowness may be attributed to the intrinsic color characteristics of lupin-derived ingredients, which typically exhibit yellow pigmentation associated with carotenoids, xanthophylls, and other naturally occurring pigments. Moreover, germination and extrusion processing can alter pigment stability, bioavailability, and interactions with macromolecular constituents of the food matrix, thereby influencing the final color attributes of the products. Feed moisture content may further affect color development by modifying heat transfer efficiency, material viscosity, residence time, and the extent of thermally induced reactions occurring during extrusion.

The pronounced responsiveness of b* to formulation and process conditions indicates that yellowness was the color attribute most strongly affected by the evaluated factors. This finding is particularly relevant for plant-based meat analogues, as visual appearance is a critical determinant of consumer perception, product acceptability, and purchase intention. Variations in yellowness may significantly influence the perceived resemblance of plant-based products to conventional meat products and, consequently, their market appeal.

The increase in b* values with increasing germinated Andean lupin whole flour incorporation may also reflect the contribution of naturally occurring pigments and lipid-associated compounds retained in the whole flour, which are largely removed during the production of highly refined protein ingredients such as soy protein isolate. Additionally, the presence of reducing sugars and free amino compounds generated during germination may have enhanced non-enzymatic browning reactions during extrusion, further contributing to the development of yellow color tones. The fitted regression model describing the behavior of b* is presented in Equation (10), and the corresponding contour plot is shown in Figure 2D.

(10) $$b^{*}=46.63+4.24x_{1}-7.44{x_{2}}^{2}$$

where b* represents the yellowness parameter of the CIELab color system, and x_1_ and x_2_ correspond to the coded values of the germinated Andean lupin whole flour–soy protein isolate ratio and feed moisture content, respectively.

**Figure 3:**
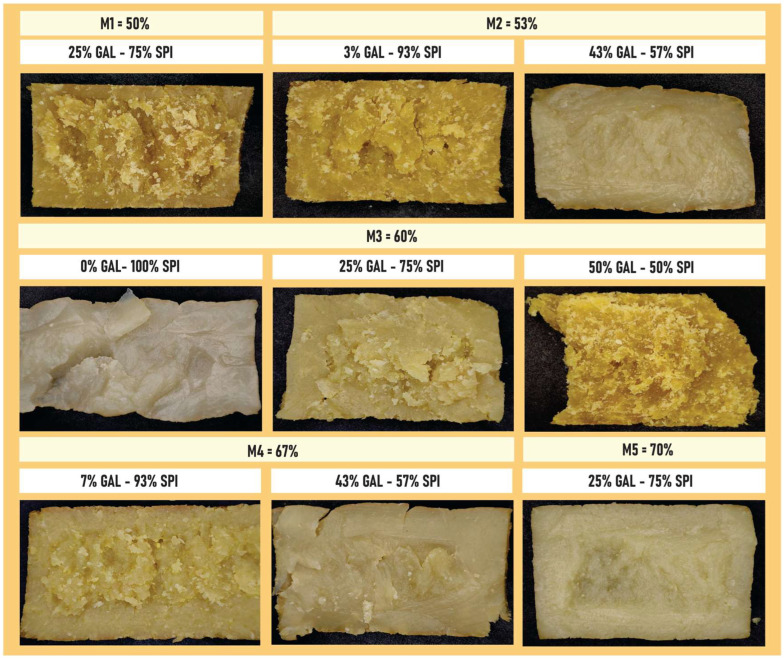
Representative cross-sectional images of extrudates from germinated Andean lupin whole flour (GAL) and soy protein isolate (SPI) blends processed at different feed moisture contents. M1–M5 correspond to feed moisture contents of 50%, 53%, 60%, 67%, and 70%, respectively.

In addition to the instrumental color measurements, the visual appearance of the extruded meat analogues is presented in Figure 3. Noticeable differences in color intensity and surface appearance were observed among the formulations, reflecting the combined influence of the germinated Andean lupin whole flour–soy protein isolate ratio and feed moisture content on product appearance. Overall, the extrudates exhibited color tones ranging from pale yellow to intense yellow, consistent with the positive b* values obtained through instrumental color analysis.

The visual differences observed among the samples corroborate the significant effects of formulation composition and processing conditions on the yellowness parameter. Formulations containing higher proportions of germinated Andean lupin whole flour generally displayed more pronounced yellow coloration, which can be attributed to the presence of naturally occurring pigments, including carotenoids and other lipid-associated chromophore compounds retained in the whole flour. Furthermore, feed moisture content may have influenced pigment stability, heat transfer dynamics, and the extent of thermally induced reactions occurring during extrusion, thereby contributing to variations in the final appearance of the products.

Beyond pigmentation effects, differences in surface morphology and light reflectance resulting from variations in extrusion conditions may also have contributed to the visual perception of color intensity. Changes in matrix structure, porosity, and surface texture can affect light scattering and absorption phenomena, influencing the apparent color of high-moisture extruded products.

The strong agreement between the instrumental measurements and visual observations indicates that b was the most responsive color parameter for distinguishing among the extruded meat analogue formulations. Accordingly, yellowness emerged as the predominant visual attribute influenced by both the incorporation of whole germinated Andean lupin flour and the high-moisture extrusion conditions. These findings highlight the importance of formulation composition and process optimization in tailoring the visual appearance of plant-based meat analogues, as visual attributes are among the primary factors influencing consumer acceptance and perceived product quality.

Color is a critical quality attribute of plant-based meat analogues because it directly influences consumer perception of quality, naturalness, and similarity to conventional meat products. In the present study, the yellowness parameter (b*) was the only color attribute significantly affected by the formulation and processing variables, indicating that the incorporation of germinated Andean lupin whole flour and feed moisture content played an important role in determining the visual appearance of the extruded products.

The increase in b* values observed with higher levels of germinated Andean lupin whole flour may be associated with the intrinsic composition of the flour. Unlike highly refined protein ingredients, whole germinated lupin flour retains endogenous pigments, lipids, phenolic compounds, and other native constituents that may contribute to the yellow coloration of the final product. Furthermore, germination promotes metabolic and enzymatic changes that modify the composition and availability of these compounds, potentially influencing their behavior during subsequent extrusion processing.

In addition to formulation effects, high-moisture extrusion can significantly affect color development through thermally induced reactions. During extrusion, proteins and carbohydrates are exposed to elevated temperatures, pressure, and mechanical shear, conditions that promote molecular transformations and the formation of colored compounds. Charles et al. [31] reported that processing variables such as barrel temperature and screw speed are important determinants of color development in extruded meat analogues. Likewise, Maillard reactions between amino groups and reducing sugars may contribute to the formation of yellow and brown pigments, thereby increasing b* values and modifying the overall appearance of the products. Köllmann et al. [32] demonstrated that reducing sugars can intensify color development in extruded meat analogues through enhanced Maillard reaction pathways.

The significant effect of feed moisture content on b* further supports the influence of processing conditions on color formation. Moisture affects heat transfer, melt viscosity, residence time, and reaction kinetics during extrusion, all of which can alter the extent of pigment degradation and non-enzymatic browning reactions. Consequently, variations in feed moisture may contribute to differences in yellowness among the extruded products.

Overall, these findings indicate that both the native composition of germinated Andean lupin whole flour and extrusion-induced thermal reactions governed the variation in b* values. From a technological standpoint, tailoring this color profile is critical to enhancing the visual appeal and consumer acceptance of meat analogues. Moreover, the natural yellow pigmentation of whole germinated Andean lupin flour is consistent with current clean-label and minimal-processing trends, providing a sustainable alternative to artificial colorants while preserving the distinctive visual appearance of plant-based meat analogues.

#### 3.2.5. Textural Properties

The textural properties of the high-moisture extruded meat analogues formulated with different germinated Andean lupin whole flour–soy protein isolate ratios and feed moisture contents are presented in Table 4. Overall, considerable variability was observed among the treatments, highlighting the combined influence of formulation composition and processing conditions on the development of the protein network during extrusion.

Hardness, chewiness, gumminess, and springiness exhibited wide variation among formulations, with values ranging from 31.68 to 457.17 N, 16.55 to 204.33 N, 32.00 to 457.75 N, and 0.806 to 0.911, respectively. However, none of the evaluated factors significantly affected these responses (*p* > 0.05), and the corresponding regression models showed limited predictive capability (R^2^ < 0.65). These findings suggest that the evaluated formulation composition and feed moisture conditions did not adequately explain the variability observed in these textural attributes, indicating that additional factors may also contribute to their development during high-moisture extrusion. Such factors may include protein alignment and molecular interactions developed during extrusion, local variations in moisture distribution, and the heterogeneous organization of the fibrous network, which are not directly captured by the experimental factors evaluated in this study [33,34]. Moreover, previous studies have shown that some texture profile analysis (TPA) parameters, particularly hardness, chewiness, gumminess, and springiness, are often influenced by the complex mechanical behavior of the extruded matrix and may be less sensitive to formulation variables alone, especially when structural differences among samples are relatively small [35]. In contrast, parameters related to structural integrity, such as cohesiveness, are generally more responsive to changes in protein network organization and therefore tend to exhibit greater predictive capability. These observations are consistent with recent studies indicating that different TPA parameters do not respond equally to formulation or processing variables because they reflect distinct mechanical aspects of the food matrix [3].

Although statistically non-significant, the observed variations indicate that intermediate feed moisture promoted the formation of denser and mechanically stronger structures, whereas higher moisture contents exerted a plasticizing effect that reduced matrix resistance. Similar behavior has been reported for high-moisture meat analogues, in which increasing feed moisture generally produces softer structures because water acts as a plasticizer, facilitating protein mobility and reducing matrix rigidity [26]. Likewise, protein denaturation, aggregation, and molecular reorganization during thermomechanical processing are recognized as key mechanisms governing texture development during high-moisture extrusion [36,37]. The ability of soy proteins to form three-dimensional networks stabilized by hydrophobic interactions and disulfide bonds may also contribute to the development of firmer structures during extrusion.

**Figure 4:**
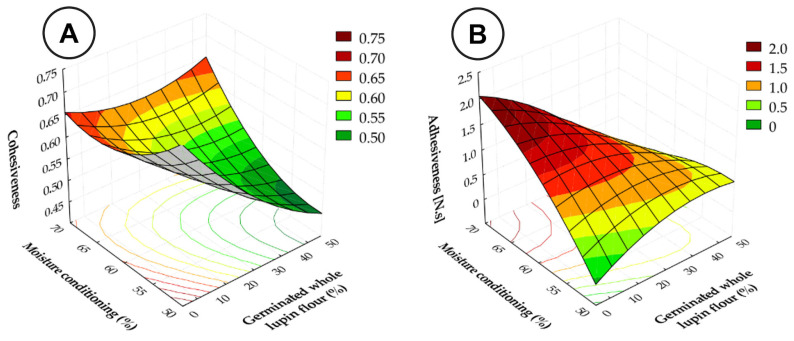
Contour plots generated from the response surface models showing the effects of the germinated Andean lupin whole flour–soy protein isolate ratio (X_1_) and feed moisture content (X_2_) on selected techno-functional properties of high-moisture extruded meat analogues: (**A**) Cohesiveness and (**B**) Adhesiveness.

In contrast, cohesiveness was significantly affected by the formulation variables and exhibited values ranging from 0.487 to 0.686. Regression analysis revealed a significant negative linear effect of germinated Andean lupin whole flour incorporation (*p* = 0.002), as well as a significant interaction between lupin flour content and feed moisture (*p* = 0.039). The fitted model showed excellent predictive performance (R^2^ = 0.91), indicating that the experimental factors adequately explained the observed variability. This higher predictive capability is expected because cohesiveness primarily depends on the continuity and integrity of the protein network, which are directly governed by protein composition and feed moisture during high-moisture extrusion. The regression model describing cohesiveness is presented in Equation (11), and the corresponding response surface is shown in Figure 4A.

(11) $$\mathrm{Cohesiveness}=0.54-0.05x_{1}+0.02{x_{1}}^{2}+0.02+0.03{x_{2}}^{2}+0.04x_{1}x_{2}$$

where x_1_ and x_2_ correspond to the coded values of the germinated Andean lupin whole flour–soy protein isolate ratio and feed moisture content, respectively.

The response surface indicated that formulations containing lower levels of germinated Andean lupin whole flour and intermediate feed moisture contents produced the highest cohesiveness values, suggesting the formation of a more continuous and integrated protein network. This observation agrees with Ramos-Díaz et al. [38], who reported that blends containing lupin protein ingredients developed well-defined fibrous structures when an adequate protein concentration and feed moisture were maintained during extrusion. Conversely, increasing lupin flour incorporation reduced cohesiveness, as evidenced by the negative linear coefficient associated with this factor.

Cohesiveness reflects the ability of a food matrix to withstand deformation and maintain structural integrity after mechanical stress. Therefore, the reduction observed by increasing lupin flour content may be attributed to the higher concentrations of dietary fiber, lipids, and non-protein components present in the whole flour. These constituents can interfere with protein–protein interactions responsible for network continuity and cohesion. Furthermore, the significant interaction between formulation and feed moisture highlights the critical role of water during extrusion, as moisture influences protein unfolding, aggregation, and molecular rearrangement. Similar trends have been reported for plant-based meat analogues, where both ingredient composition and extrusion conditions strongly affected cohesiveness and structural organization [39,40].

Adhesiveness ranged from 0.30 to 1.93 N·s and was also significantly influenced by the formulation variables. Regression analysis revealed a significant linear effect of germinated Andean lupin whole flour incorporation (*p* = 0.011) and a significant interaction between lupin flour content and feed moisture (*p* = 0.017), while the linear moisture term showed a tendency toward significance (*p* = 0.057). The fitted model demonstrated satisfactory predictive performance (R^2^ = 0.88). The regression model describing adhesiveness is presented in Equation (12), and the corresponding contour plot is shown in Figure 4B.

(12) $$\mathrm{Adhesiveness}=1.14-0.31x_{1}-0.15{x_{1}}^{2}+0.20x_{2}-0.15{x_{2}}^{2}-0.40x_{1}x_{2}$$

where adhesiveness (N·s) is the predicted response, and x_1_ and x_2_ correspond to the coded values of the germinated Andean lupin whole flour–soy protein isolate ratio and feed moisture content, respectively.

The response surface indicated that higher adhesiveness values were obtained at higher feed moisture contents and lower levels of germinated Andean lupin whole flour. Conversely, increasing lupin flour incorporation reduced adhesiveness, consistent with the negative linear coefficient observed in the model.

Adhesiveness represents the work required to overcome the attractive forces between the sample surface and the probe during texture profile analysis and is closely associated with free water availability and matrix hydration properties. Under high-moisture extrusion conditions, water acts as a plasticizer, increasing molecular mobility and facilitating protein network reorganization, which directly influences the adhesive behavior of the extruded matrix. However, excessive water may also increase surface moisture retention, resulting in greater adhesion between the product and the probe. The reduction in adhesiveness associated with increasing lupin flour content may be related to the high dietary fiber content of the flour, which modifies water distribution within the matrix and reduces the amount of free water available at the product surface. Similar effects have been reported in plant-based meat analogues, where adhesiveness was strongly influenced by both ingredient composition and extrusion conditions [40,41,42].

Despite the reduction in cohesiveness and adhesiveness at higher lupin incorporation levels, the textural properties remained within the range reported for commercial and experimental high-moisture meat analogues, demonstrating the feasibility of partially replacing highly refined soy protein isolate with a minimally processed germinated ingredient.

### 3.3. Response Surface Modeling, Numerical Optimization, and Validation of the Optimized Extruded Meat Analog

A multi-response numerical optimization was performed using the desirability function approach to identify the optimal formulation and processing conditions to produce high-moisture extruded meat analogues. Only responses with statistically significant predictive models (*p* < 0.05, F_calc_ > F_tab_, and R^2^ > 0.75) were included in the optimization procedure. Each response was assigned an importance value ranging from 1 (lowest importance) to 5 (highest importance) according to its technological relevance.

The optimization criteria were defined to maximize OAC, cohesiveness, and adhesiveness, while minimizing the WSI and CL. The instrumental color parameter b* was maintained within the experimental range to preserve an acceptable visual appearance without imposing a specific optimization target.

Based on these criteria, the optimal formulation consisted of 12% germinated Andean lupin whole flour, 88% soy protein isolate, and a feed moisture content of 65.5%, resulting in an overall desirability of 77.82%. This desirability value indicates a satisfactory compromise among the different technological responses, demonstrating that a 12% substitution level provides the best technological compromise among the evaluated responses, while supporting the incorporation of a minimally processed germinated ingredient into high-moisture meat analogues.

The present results indicate that GAL acts primarily as a complementary protein ingredient and nutritional enhancer rather than as a substitute for substantial SPI replacement. Although GAL contains approximately 45% protein, it is a whole flour that also provides dietary fiber, endogenous lipids, minerals, and other non-protein constituents, whereas SPI contains approximately 88% protein and forms a more concentrated protein phase during high-moisture extrusion. Consequently, increasing GAL incorporation reduces the proportion of highly concentrated network-forming protein and may disrupt protein aggregation, cross-linking, and molecular alignment because of the presence of fiber and other non-protein components [43,44,45].

Despite this limitation, the optimized 12% GAL inclusion remains practically relevant. Previous studies have demonstrated that partial substitution, rather than complete replacement, is generally the most feasible strategy for incorporating alternative plant proteins into high-moisture meat analogues while maintaining desirable structural and functional properties [46,47,48]. From an industrial perspective, this substitution level may also represent a considerable volume of ingredient use. For example, in a production facility processing 10 metric tons of dry protein blend per day, a 12% GAL inclusion would require approximately 1.2 metric tons of GAL daily, replacing an equivalent amount of SPI. Thus, although the optimized inclusion level is numerically moderate, it represents a meaningful industrial incorporation of a minimally processed Andean ingredient while preserving the technological performance of the final product.

The predicted values generated by the optimization procedure, together with the corresponding experimental values obtained during model validation, are presented in Table 5. The close agreement between predicted and experimental results confirms the adequacy of the selected models and supports the reliability of the optimization strategy for predicting the techno-functional properties of the extruded products. Notably, the optimized formulation achieved the desired technological performance while replacing 12% of the soy protein isolate with a minimally processed germinated ingredient, highlighting the potential of germinated Andean lupin whole flour as a sustainable alternative for the development of next-generation plant-based meat analogues.

To validate the optimized condition, three independent extrusion runs were performed under the predicted optimal processing conditions, and the experimental responses were compared with those estimated by the mathematical models. Relative deviations between predicted and experimental values were lower than 10% for all responses, demonstrating the adequacy and predictive capability of the models for describing the technological properties of extruded meat analogues within the evaluated experimental domain.

These findings demonstrate that combining germinated Andean lupin whole flour with soy protein isolate at the optimized formulation provides a balanced technological performance, characterized by improved oil absorption capacity, desirable textural properties, reduced water solubility and cooking loss, and acceptable color attributes. Therefore, GAL can be considered a promising complementary protein ingredient for partially replacing soy protein isolate while maintaining the technological quality required for high-moisture meat analogues. Under the evaluated conditions, its main contribution lies in ingredient diversification and nutritional enhancement rather than substantial replacement of soy protein isolate.

Although the present study was conducted at laboratory scale, the optimized formulation was produced using high-moisture extrusion, a technology already established at industrial scale for manufacturing plant-based meat analogues. Therefore, the optimized processing conditions provide a technically feasible basis for future pilot- and industrial-scale validation. However, additional studies are required to evaluate process robustness, production consistency, and product quality under continuous manufacturing conditions.

### 3.4. Nutritional Characterization of the Optimized Extruded Meat Analog

#### 3.4.1. Proximate Composition

The technological and nutritional characteristics of the optimized meat analogue formulation identified through the desirability function and subsequently validated experimentally are presented in Table 6. The selected formulation, consisting of 12% germinated Andean lupin whole flour, 88% soy protein isolate, and 65.5% feed moisture content, was subjected to comprehensive characterization, including proximate composition, in vitro protein digestibility, amino acid profile, techno-functional properties, instrumental color parameters, and texture profile analysis. These results provide a detailed assessment of the nutritional quality and technological performance of the optimized high-moisture extruded meat analogue.

The optimized meat analogue exhibited a remarkably high protein content (84.45%), accompanied by low lipid (1.12%) and ash (4.12%) contents, highlighting its potential as a nutrient-dense, protein-rich plant-based food. The high protein content can be primarily attributed to the predominance of soy protein isolate in the optimized formulation, complemented by the incorporation of germinated Andean lupin whole flour, which provides additional proteins, dietary fiber, and bioactive compounds generated during germination. These findings demonstrate that the partial replacement of SPI with a minimally processed plant ingredient can be achieved without compromising the overall protein density of the final product, thereby supporting the development of more sustainable and less refined protein-based formulations.

From a technological standpoint, the optimized meat analogue exhibited favorable hydration and lipid-binding properties, as evidenced by its WAI and OAC. These functional attributes are particularly important in plant-based meat systems because they directly influence flavor retention, juiciness, mouthfeel, cooking performance, and product stability throughout processing and consumption. Improved water and oil retention capacities have been associated with enhanced sensory quality, greater moisture retention, and reduced perception of dryness in plant-based meat products [49]. Moreover, adequate hydration properties facilitate molecular mobility during extrusion and contribute to the development of fibrous structures and desirable textural characteristics in high-moisture protein systems [50].

**Figure 5:**
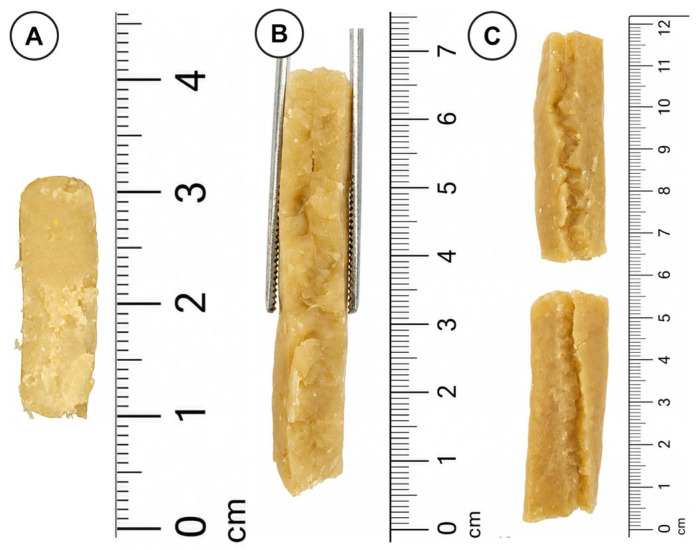
Macroscopic appearance and longitudinal opening behavior of the optimized high-moisture extruded product. (**A**) Cross-sectional view of the optimized extrudate; (**B**) longitudinal view showing the exposed internal matrix; and (**C**) longitudinally separated segments illustrating the internal opening and fracture behavior. The images provide qualitative evidence of a cohesive and directionally structured matrix; fiber alignment and anisotropy were not quantitatively determined.

Texture profile analysis further supported the technological suitability of the optimized formulation. The observed hardness, cohesiveness, and springiness values indicate the development of a cohesive, resilient, and elastic protein matrix capable of maintaining structural integrity under mechanical deformation. Representative photographs of the optimized extrudate are shown in Figure 5. Visual inspection revealed a compact internal matrix with irregular layered regions and an apparent directional organization along the extrusion axis. The longitudinally separated samples also exhibited a non-uniform fracture pattern, suggesting the development of structured domains during high-moisture extrusion. These macroscopic observations are consistent with the instrumental texture results and may be associated with protein denaturation, unfolding, molecular rearrangement, aggregation, and intermolecular interactions occurring during processing.

The high protein content achieved in the present study is comparable to, and in some cases exceeds, values reported for other plant-based meat analogues formulated with soy proteins combined with emerging plant protein sources. Song et al. [51] demonstrated that hybrid protein systems can simultaneously improve nutritional quality and technological functionality through complementary amino acid profiles and enhanced structuring behavior during extrusion. Similarly, recent reviews have emphasized that one of the major challenges in plant-based meat development is achieving an optimal balance between nutritional value, functionality, and meat-like texture. In this context, high-moisture extrusion remains one of the most effective technologies for generating directionally structured protein matrices with textural attributes relevant to plant-based meat analogues [52,53].

Beyond its technological and nutritional advantages, the incorporation of whole germinated Andean lupin flour represents a sustainable strategy for valorizing an underutilized Andean legume with exceptional nutritional potential. This approach is consistent with current efforts to diversify protein sources in plant-based foods while reducing reliance on highly refined protein ingredients. In addition, germination enhances the nutritional quality of lupin through the accumulation of bioactive compounds, the reduction in antinutritional factors, and favorable modifications to its biochemical composition, thereby increasing the functional value of the resulting meat analogue.

Overall, the results demonstrate that germinated Andean lupin whole flour is a promising ingredient for developing sustainable and high-protein meat analogues produced by high-moisture extrusion. Its successful incorporation into the optimized formulation highlights the feasibility of partially replacing highly refined protein ingredients while maintaining desirable nutritional, functional, and textural characteristics. This strategy is fully aligned with current industry and consumer demands for clean-label products, ingredient diversification, reduced processing intensity, and more sustainable plant-based food systems.

#### 3.4.2. Amino Acid Profile and In Vitro Protein Digestibility

The total amino acid (TAA) profile and in vitro protein digestibility of the soy protein isolate, germinated Andean lupin whole flour, and control and optimal point meat analogue are presented in Table 7.

**Figure 6:**
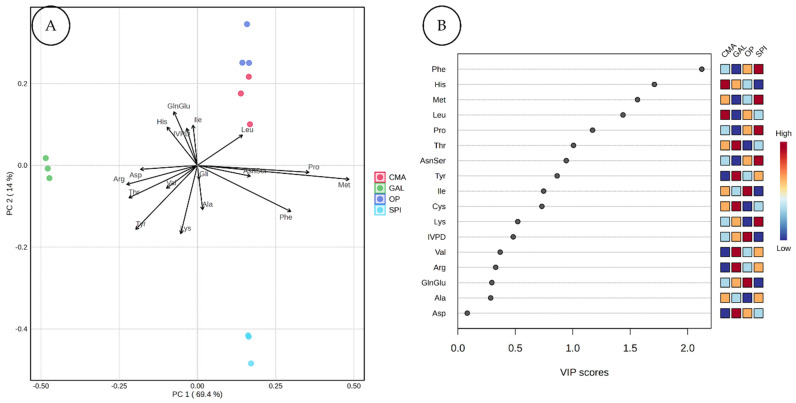
Multivariate analysis of amino acid composition and in vitro protein digestibility (IVPD) of soy protein isolate (SPI), germinated Andean lupin whole flour (GAL), control high-moisture meat analogue (CMA), and optimized high-moisture meat analogue (OP): (**A**) principal component analysis (PCA) biplot and (**B**) variable importance in projection (VIP) scores obtained from partial least squares discriminant analysis (PLS-DA). Phe = phenylalanine; His = histidine; Met = methionine; Leu = leucine; Pro = proline; Thr = threonine; AsnSer = asparagine + serine + hydroxyproline; Tyr = tyrosine; Ile = isoleucine; Cys = cysteine; Lys = lysine; Val = valine; Arg = arginine; GlnGlu = glutamine + glutamic acid; Ala = alanine; Asp = aspartic acid.

Complementary insights regarding compositional variability and the principal variables driving group discrimination were provided by multivariate analyses combining PCA (Figure 6A) and PLS-DA variable importance in projection (VIP) scores (Figure 6B). Overall, it was demonstrated that the partial replacement of soy protein isolate with germinated Andean lupin whole flour improved the amino acid profile of the optimized formulation and enhanced protein digestibility, thereby supporting the nutritional potential of germinated Andean lupin flour for high-moisture extruded meat analogues.

Total essential amino acids (EAAs) varied among samples, with values of 36.98 g·100 g^−1^ protein for soy protein isolate, 33.09 g·100 g^−1^ protein for germinated whole Andean lupin flour, 39.99 g·100 g^−1^ protein for control meat analogue, and 33.32 g·100 g^−1^ protein for optimal point meat analogue. Although optimal point meat analogue showed a lower EAA content than control meat analogue, it maintained nutritionally relevant levels of most essential amino acids, particularly leucine (8.21 g·100 g^−1^ protein), lysine (5.17 g·100 g^−1^ protein), isoleucine (4.76 g·100 g^−1^ protein), valine (4.37 g·100 g^−1^ protein), and phenylalanine (3.69 g·100 g^−1^ protein). The EAA/TAA ratio was 39.72% for soy protein isolate, 34.40% for germinated whole Andean lupin flour, 43.85% for control meat analogue, and 34.81% for optimal point meat analogue, indicating that approximately one-third of the total amino acids in the optimized product corresponded to essential amino acids. This result is nutritionally relevant because protein quality in plant-based meat analogues depends not only on total protein content but also on the balance and availability of essential amino acids after digestion [54,55].

The branched-chain amino acid (BCAA) fraction, calculated as leucine + isoleucine + valine, was 16.84 g·100 g^−1^ protein in soy protein isolate, 16.36 g·100 g^−1^ protein in germinated whole Andean lupin flour, 22.36 g·100 g^−1^ protein in control meat analogue, and 17.34 g·100 g^−1^ protein in optimal point meat analogue. The optimized meat analogue therefore retained a high BCAA content, mainly due to its leucine concentration. Leucine is particularly important because it is involved in the regulation of skeletal muscle protein synthesis through the mTOR (mechanistic Target of Rapamycin) signaling pathway, while isoleucine and valine contribute to nitrogen balance and muscle metabolism [56,57]. From a nutritional perspective, the BCAA content of optimal point meat analogue supports its potential as a high-protein plant-based product.

Regarding sulfur-containing amino acids (SAA), calculated as methionine + cysteine, soy protein isolate presented 3.15 g·100 g^−1^ protein, germinated whole Andean lupin flour 1.51 g·100 g^−1^ protein, control meat analogue 2.91 g·100 g^−1^ protein, and optimal point meat analogue 2.71 g·100 g^−1^ protein. The low methionine content of germinated whole Andean lupin flour (0.17 g·100 g^−1^ protein) confirms that sulfur amino acids are limiting in germinated Andean lupin flour. However, the optimized meat analogue exceeded the adult amino acid requirement pattern proposed by FAO (Food and Agriculture Organization) of the United Nations for total sulfur amino acids, demonstrating that the combination with soy protein isolate compensated for this limitation. The aromatic amino acid fraction, calculated as phenylalanine + tyrosine, was 8.91, 6.26, 5.36, and 6.31 g·100 g^−1^ protein for the soy protein isolate, germinated whole Andean lupin flour, control meat analogue, and optimal point meat analogue, respectively. Thus, the optimal point meat analogue showed a 17.7% higher aromatic amino acid content than the control meat analogue, mainly due to the increase in phenylalanine.

When compared with the FAO adult amino acid requirement pattern, optimal point meat analogue met or exceeded the recommended levels for histidine, isoleucine, leucine, lysine, sulfur amino acids, aromatic amino acids, and valine. Threonine was the only limiting essential amino acid in the optimized formulation, with 1.94 g·100 g^−1^ protein, corresponding to approximately 84% of the FAO adult reference value. Nevertheless, threonine remained the limiting indispensable amino acid in the optimized formulation according to the FAO adult amino acid reference pattern. Although this limitation can be effectively addressed through complementary consumption with cereal proteins or other threonine-rich plant ingredients, a strategy widely adopted in plant-based food formulations to achieve balanced amino acid profiles [54,55], it should be acknowledged when evaluating the nutritional quality of the product. Overall, the optimized meat analogue provided adequate levels of the remaining essential amino acids, together with a high branched-chain amino acid content and excellent in vitro protein digestibility. Therefore, the formulation represents a nutritionally valuable complementary plant protein ingredient with considerable potential for incorporation into balanced plant-based diets.

The PCA biplot provided additional evidence of the nutritional differences among the evaluated matrices. The first principal component (PC1) explained 69.4% of the total variance, and the second principal component (PC2) explained 14.0%, accounting for 83.4% of the total variability. Germinated whole Andean lupin flour was clearly separated from soy protein isolate and the extruded meat analogues, showing stronger associations with arginine, aspartic acid, lysine, threonine, and tyrosine. This behavior reflects the characteristic amino acid profile of lupin proteins, particularly their high arginine content. In fact, germinated whole Andean lupin flour showed the highest arginine concentration (12.19 g·100 g^−1^ protein), while the optimal point meat analogue contained 7.61 g·100 g^−1^ protein, representing a 25.8% increase compared with the control meat analogue. In contrast, soy protein isolate and the meat analogues were more closely associated with methionine, proline, phenylalanine, leucine, and asparagine/serine, reflecting the predominance of soybean proteins and the influence of extrusion-induced protein restructuring.

The separation of the optimal point meat analogue from the control meat analogue in the PCA indicates that even a 12% incorporation of germinated whole Andean lupin flour was sufficient to alter the nutritional fingerprint of the final product. This effect was particularly evident for glutamic acid + glutamine and serine + asparagine + hydroxyproline, which increased from 14.05 to 18.20 g·100 g^−1^ protein and from 6.05 to 11.85 g·100 g^−1^ protein, respectively, when comparing the control meat analogue and optimal point meat analogue. These increases suggest that germinated whole Andean lupin flour contributed not only essential amino acids but also nitrogenous compounds associated with taste, protein functionality, and matrix interactions. Germination may have contributed to these changes by activating endogenous proteases, promoting partial hydrolysis of storage proteins, and increasing the concentration of free amino acids and low-molecular-weight peptides [2,58,59].

PLS-DA further supported the exploratory trends observed in PCA and identified the amino acids most responsible for sample discrimination. Phenylalanine showed the highest VIP score (>2.0), followed by histidine, methionine, leucine, proline, threonine, and asparagine/serine. The presence of several essential amino acids among the most discriminant variables indicates that the differences among soy protein isolate, germinated whole Andean lupin flour, control meat analogue, and optimal point meat analogue were strongly related to protein quality. The high VIP scores for methionine and leucine are especially relevant because sulfur amino acids and BCAA are nutritionally critical in plant-based protein systems.

It should be noted that the PCA and PLS-DA analyses were performed as exploratory chemometric tools to facilitate the interpretation of differences among protein sources and extruded products. Considering the relatively limited number of samples included in the optimization study, the PLS-DA results and VIP scores should be interpreted with caution, as additional validation procedures (e.g., cross-validation, permutation testing, and external validation) would be required to establish the predictive performance and stability of the discriminant model.

IVPD values ranged from 79.3% in soy protein isolate to 90.1% in the optimal point meat analogue. Germinated whole Andean lupin flour presented higher digestibility (88.3%) than soy protein isolate, suggesting that germination improved protein susceptibility to enzymatic hydrolysis. Crucially, the optimized formulation exhibited the highest IVPD value, outperforming the control meat analogue by 4.5 percentage points and the raw soy protein isolate by 10.8 percentage points. This nutritional enhancement stems from the synergistic effects of germination and high-moisture extrusion. While germination mitigates antinutritional factors, such as phytic acid and protease inhibitors, extrusion induces protein denaturation, unfolding, and structural reorganization, enhancing the accessibility of peptide bonds to digestive proteases [15,60,61].

Moreover, the slight limitation observed for threonine does not substantially compromise the nutritional quality of the product, as complementary protein strategies are well established in plant-based food formulation and can readily provide a balanced essential amino acid profile. Although threonine remained the limiting amino acid, the optimal point meat analogue exceeded the FAO adult requirements for most essential amino acids and showed high levels of BCAA, sulfur amino acids, aromatic amino acids, and arginine. Therefore, the partial incorporation of germinated Andean lupin flour into a soy-based matrix represents a promising strategy to diversify the protein profile, improve digestibility, and reduce dependence on highly refined protein ingredients in next-generation plant-based meat analogues.

Although the optimized formulation exhibited a high IVPD (90.1%), these results should be interpreted considering the inherent limitations of in vitro digestion assays. Simulated gastrointestinal digestion provides a rapid, standardized, and reproducible estimate of protein susceptibility to enzymatic hydrolysis and is widely used to compare protein ingredients and processing technologies. Nevertheless, IVPD does not fully reproduce the complexity of human digestion and therefore cannot be directly extrapolated to amino acid bioavailability or physiological protein utilization. Factors such as gastric emptying, intestinal transit, endogenous enzyme secretion, amino acid transport, gut microbiota interactions, and post-absorptive metabolism are not represented in static in vitro models. Consequently, the improved digestibility observed in the optimized meat analogue should be interpreted as evidence of enhanced enzymatic accessibility resulting from germination and high-moisture extrusion rather than definitive confirmation of superior nutritional utilization in vivo. Future studies employing standardized ileal digestibility methods (DIAAS) or controlled in vivo trials are warranted to validate the nutritional benefits suggested by the present findings [2,61,62].

Although anti-nutritional compounds such as phytates and protease inhibitors were not quantified in the present study, the germinated Andean lupin whole flour used for meat analogue production was obtained under germination conditions previously optimized by our research group, which significantly improved protein digestibility and nutritional quality [2]. Furthermore, the Lupinus mutabilis cultivar employed is characterized by a low alkaloid content, minimizing concerns regarding quinolizidine alkaloids. Nevertheless, quantifying residual phytates and protease inhibitors after germination and high-moisture extrusion would provide a more comprehensive understanding of the nutritional quality of the ingredient and should be addressed in future studies. It should also be noted that the optimized extruded material developed in this work is intended as an intermediate food ingredient rather than a ready-to-eat product and will undergo subsequent processing or incorporation into plant-based food formulations before consumption.

## 4. Conclusions

This study demonstrated the technical feasibility of partially replacing soy protein isolates with germinated Andean lupin whole flour to produce high-moisture extruded meat analogues. The incorporation of germinated Andean lupin whole flour significantly influenced key techno-functional properties, particularly the water solubility index, oil absorption capacity, cooking loss, cohesiveness, adhesiveness, and yellowness (b*), generating predictive models with satisfactory goodness-of-fit and enabling the optimization of formulation and processing conditions through the Response Surface Methodology. The optimized formulation, consisting of 12% germinated Andean lupin whole flour, 88% soy protein isolate, and 65.5% feed moisture content, achieved a global desirability of 77.82% and was successfully validated experimentally.

The optimized meat analogue exhibited favorable technological performance, including low cooking loss, adequate hydration and lipid-binding properties, and desirable textural characteristics associated with the formation of a cohesive and structurally stable protein network during high-moisture extrusion. In addition, the product showed a remarkably high protein content (84.44%), enhanced protein quality, improved essential amino acid composition, increased branched-chain amino acid levels, and superior in vitro protein digestibility compared with the control formulation. These findings demonstrate that incorporating a minimally processed germinated legume ingredient can improve the nutritional profile of plant-based meat analogues while maintaining the structural and functional requirements necessary for successful extrusion processing.

Beyond its technological and nutritional advantages, the use of germinated Andean lupin whole flour contributes to the valorization of underutilized Andean legumes and supports the development of more sustainable and less refined plant-based food systems. The present findings demonstrate that germinated Andean lupin whole flour is a promising complementary protein ingredient for partially replacing soy protein isolate while preserving the technological performance required for high-moisture extrusion.

Future research should focus on incorporating the optimized meat analogue into ready-to-eat plant-based products, such as plant-based sausages, followed by comprehensive sensory evaluation to assess texture, flavor, mouthfeel, and overall consumer acceptance under realistic consumption conditions. Additional studies should also investigate the molecular mechanisms governing fibrous structure formation and the bioavailability of nutrients and bioactive compounds to further support industrial application.

## Figures and Tables

**Table 1:** Coded and actual levels of the central composite design used for the development of high-moisture extruded meat analogues formulated with germinated Andean lupin whole flour and soy protein isolate.

Trial	Coded Levels		Real Levels			
	x_1_	x_2_	X_1_ (%)			X_2_ (%)
1	−1	−1	7	:	93	53
2	1	−1	43	:	57	53
3	−1	1	7	:	93	67
4	1	1	43	:	57	67
5	−1.41	0	0	:	100	60
6	1.41	0	50	:	50	60
7	0	−1.41	25	:	75	50
8	0	1.41	25	:	75	70
9	0	0	25	:	75	60
10	0	0	25	:	75	60
11	0	0	25	:	75	60

where x_1_ and X_1_ represent the coded and actual levels, respectively, of the germinated Andean lupin whole flour-to-soy protein isolate ratio, whereas x_2_ and X_2_ represent the coded and actual levels, respectively, of feed moisture conditioning.

**Table 2:** Techno-functional properties of high-moisture extruded meat analogues as affected by the germinated Andean lupin whole flour–soy protein isolate ratio and feed moisture content.

Trials	WAI (%)	WSI (%)	OAC (%)	CRC (%)	CL (%)
1	4.28 ± 0.02	7.78 ± 0.31	175.13 ± 5.49	15.04 ± 1.07	1.41 ± 0.06
2	3.97 ± 0.17	18.79 ± 0.94	166.57 ± 1.06	16.76 ± 1.59	2.57 ± 0.01
3	4.23 ± 0.05	6.85 ± 0.07	190.82 ± 6.62	8.46 ± 0.41	1.23 ± 0.11
4	4.19 ± 0.12	16.77 ± 0.46	179.91 ± 0.61	24.50 ± 1.54	2.54 ± 0.06
5	5.62 ± 1.09	6.21 ± 0.31	185.71 ± 2.87	12.45 ± 0.29	1.01 ± 0.08
6	4.00 ± 0.21	18.03 ± 0.46	180.50 ± 4.65	81.53 ± 1.51	3.84 ± 0.63
7	3.72 ± 0.59	11.69 ± 2.09	172.37 ± 9.30	43.79 ± 7.58	2.40 ± 0.38
8	4.65 ± 0.71	7.78 ± 0.80	239.42 ± 12.69	10.31 ± 0.47	1.38 ± 0.07
9	4.20 ± 0.16	12.16 ± 1.24	202.50 ± 6.77	9.77 ± 0.09	1.92 ± 0.21
10	4.32 ± 0.23	12.25 ± 1.09	204.58 ± 12.23	11.70 ± 0.63	1.90 ± 0.06
11	4.15 ± 0.09	12.07 ± 4.36	206.61 ± 2.26	12.25 ± 1.52	1.79 ± 0.08
*p*-value	0.256	0.002	0.001	0.438	0.001
R^2^	0.6506	0.9588	0.9330	0.5366	0.9060
R^2^-adjusted	0.3013	0.9177	0.8882	0.1870	0.8657
Lack of fit	0.039	0.154	0.123	0.003	0.386

Values are expressed as mean ± standard deviation (n = 3). WAI = Water Absorption Index (%); WSI = Water Solubility Index (%); OAC = Oil Absorption Capacity (%); CRC = Cooking Rehydration Capacity (%); CL = Cooking Loss (%); R^2^ = Regression Determination.

**Table 3:** Instrumental color parameters (L*, a*, and b*) and corresponding visual appearance of high-moisture extruded meat analogues as affected by the germinated Andean lupin whole flour–soy protein isolate ratio and feed moisture content.

Formulation	Parameter L*	Parameter a*	Parameter b*	Estimate Color ^§^
1	65.95 ± 1.06	8.05 ± 0.05	30.85 ± 0.43	
2	75.88 ± 0.47	6.56 ± 0.10	44.55 ± 0.21	
3	75.88 ± 1.28	7.20 ± 0.71	35.31 ± 0.49	
4	78.55 ± 1.35	7.54 ± 0.55	45.32 ± 0.76	
5	80.36 ± 2.55	5.09 ±0.74	38.89 ± 0.30	
6	80.38 ± 1.80	4.67 ± 0.07	46.07 ± 1.03	
7	74.36 ± 3.08	6.94 ± 0.80	34.82 ± 0.70	
8	84.79 ± 0.64	3.62 ± 0.01	26.33 ± 0.16	
9	77.53 ± 0.42	6.53 ± 0.18	42.20 ± 0.55	
10	81.86 ± 1.35	5.28 ± 0.37	50.17 ± 0.76	
11	69.23 ± 1.76	4.74 ± 0.53	47.56 ± 0.29	
*p*-value	0.622	0.868	0.002	
R^2^	0.4271	0.2546	0.7783	
R^2^-adjusted	<0.0001	<0.0001	0.7529	
Lack of fit	0.622	0.166	0.596	

Mean of three repetitions + standard deviation. ^§^ Visual representations of the Nix™ color sensor are available at <https://www.nixsensor.com/free-color-converter/> (accessed at 30 March 2026).

**Table 4:** Texture profile analysis (TPA) parameters of high-moisture extruded meat analogues formulated with germinated Andean lupin whole flour and soy protein isolate.

Trial	Hardness (N)	Cohesiveness	Elasticity	Chewability (N)	Gumminess (N)	Adhesiveness (N·s)
1	62.09 ± 1.67	0.686 ± 0.03	0.866 ± 0.013	36.96 ± 3.66	62.42 ± 24.95	0.42 ± 1.25
2	279.60 ± 12.60	0.503 ± 0.05	0.849 ± 0.011	118.93 ± 12.68	279.80 ± 2.29	0.65 ± 0.36
3	191.26 ± 8.56	0.615 ± 0.03	0.872 ± 0.009	102.22 ± 3.51	189.29 ± 8.46	1.93 ± 0.33
4	73.24 ± 15.29	0.581 ± 0.06	0.806 ± 0.010	34.22 ± 10.47	73.86 ± 6.11	0.56 ± 1.28
5	230.90 ± 4.05	0.639 ± 0.03	0.872 ± 0.005	128.68 ± 4.13	233.54 ± 3.86	1.27 ± 0.90
6	457.17 ± 12.50	0.487 ± 0.03	0.905 ± 0.102	201.74 ± 21.23	457.75 ± 16.59	0.30 ± 0.47
7	422.18 ± 3.97	0.531 ± 0.06	0.911 ± 0.084	204.33 ± 12.95	423.23 ± 27.60	0.75 ± 1.05
8	31.68 ± 5.93	0.626 ± 0.05	0.838 ± 0.036	16.55 ± 3.25	32.00 ± 5.86	0.85 ± 0.41
9	159.54 ± 10.43	0.525 ± 0.05	0.861 ± 0.005	71.34 ± 6.89	159.75 ± 25.31	1.17 ± 0.67
10	111.08 ± 14.41	0.550 ± 0.03	0.853 ± 0.007	51.76 ± 4.25	111.35 ± 14.99	1.02 ± 0.76
11	153.75 ± 11.01	0.541 ± 0.02	0.855 ± 0.003	71.21 ± 6.75	153.80 ± 11.18	1.22 ± 0.88
*p*-value	0.277	0.012	0.628	0.364	0.272	0.022
R^2^	0.6364	0.9096	0.4237	0.5813	0.6358	0.8836
R^2^-adjusted	0.2728	0.8193	<0.0001	0.1626	0.2716	0.7671
Lack of fit	0.029	0.132	0.012	0.022	0.029	0.122

Values are expressed as mean ± standard deviation (n = 6). Hardness (N), Chewability (N), Gumminess (N), and Adhesiveness (N·s) were determined by Texture Profile Analysis (TPA). Cohesiveness and Elasticity are dimensionless parameters.

**Table 5:** Predicted and experimental responses, relative deviation, and validation of the multi-response optimization model for high-moisture extruded meat analogues.

Independent Variables							
Parameters	Goal	Lower Limit	Upper Limit	Importance	Solution		
					Coded Value	Real Value	
Proportion of germinated Andean lupin whole flour:soy protein isolate	In range	−1.41	1.41	3	−0.73	12:88	
Feed moisture conditioning (%)	In range	−1.41	1.41	3	0.77	65.50	
**Dependent Variables**							
**Parameters**	**Goal**	**Lower Limit**	**Upper Limit**	**Importance**	**Predicted Value**	**Experimental Validation Value**	**Relative Deviation (%)**
Cooking Loss (%)	Minimize	1.01	3.84	5	1.20	1.22 ± 0.09	1.67
Water Solubility Index (%)	Minimize	6.21	18.79	5	7.75	7.29 ± 0.32	5.94
Oil Absorption Capacity (%)	Maximize	166.57	206.61	5	194.53	207.25 ± 7.89	6.54
Cohesiveness	Maximize	0.49	0.69	5	0.60	0.65 ± 0.03	8.33
Adhesiveness (N·s)	Maximize	0.30	1.92	5	1.58	1.45 ± 0.11	8.22
b*	In range	26.33	50.17	5	38.51	37.49 ± 2.19	2.65
Desirability 77.82%							

For the desirability analysis, lower and upper weights were set to 1 for all variables, corresponding to linear desirability functions.

**Table 6:** Physicochemical, techno-functional, color, and textural properties of the optimized high-moisture extruded meat analogue formulated with germinated Andean lupin whole flour and soy protein isolate.

Properties	Optimal High-Moisture Meat Analogue
Proximal composition ^#^	
Protein	84.45 ± 0.05
Fat	1.12 ± 0.14
Ash	4.12 ± 0.22
Carbohydrates	10.31 ± 0.08
Technofunctional properties ^#^	
Cooking rehydration capacity (%)	12.89 ± 1.73
Cooking loss (%)	1.22 ± 0.09
Water absorption index (%)	5.2 ± 0.21
Water solubility index (%)	7.29 ± 0.32
Oil absorption capacity (%)	207.25 ± 7.89
Instrumental color ^#^	
Parameter L*	80.85 ± 0.45
Parameter a*	4.64 ± 0.13
Parameter b*	37.49 ± 2.19
Instrumental texture ^£^	
Hardness (N)	55.35 ± 3.17
Cohesiveness	0.65 ± 0.03
Elasticity	0.97 ± 0.20
Chewability (N)	40.33 ± 6.35
Gumminess (N)	56.07 ± 3.14
Adhesiveness (N.s)	1.45 ± 0.11

Values are expressed as mean ± standard deviation. **^#^** Values obtained from three repetitions. ^£^ Values obtained from six repetitions.

**Table 7:** Essential and non-essential amino acid profiles (g·100 g^−1^ protein, d.b.) and in vitro protein digestibility (%) of soy protein isolate, germinated Andean lupin whole flour, control meat analogue, and optimized high-moisture extruded meat analogue.

Response	Soy Protein Isolate	Germinated Andean Lupin Whole Flour	Control High-Moisture Extruded Meat Analogue	Optimal Point of High-Moisture Extruded Meat Analogue
Essential amino acids				
Histidine	3.29 ± 0.03	4.50 ± 0.05	4.53 ± 0.17	3.65 ± 0.52
Isoleucine	4.18 ± 0.02	4.63 ± 0.25	5.07 ± 0.07	4.76 ± 0.02
Leucine	8.06 ± 0.02	6.80 ± 0.59	13.10 ± 0.32	8.21 ± 0.25
Lysine	7.52 ± 0.34	6.83 ± 0.03	6.09 ± 0.15	5.17 ± 0.42
Methionine	1.91 ± 0.01	0.17 ± 0.02	1.58 ± 0.05	1.53 ± 0.28
Phenylalanine	5.07 ± 0.04	1.49 ± 0.09	2.96 ± 0.04	3.69 ± 0.09
Threonine	2.35 ± 0.01	3.74 ± 0.07	2.47 ± 0.10	1.94 ± 0.16
Valine	4.60 ± 0.01	4.93 ± 0.06	4.19 ± 0.05	4.37 ± 0.12
Non-essential amino acids				
Alanine	4.82 ± 0.02	4.24 ± 0.11	4.73 ± 0.02	3.54 ± 0.07
Arginine	7.80 ± 0.01	12.19 ± 0.44	6.05 ± 0.33	7.61 ± 0.02
Aspartic acid	6.62 ± 0.16	9.19 ± 0.84	6.11 ± 0.22	6.81 ± 0.01
Cysteine	1.24 ± 0.01	1.34 ± 0.12	1.33 ± 0.03	1.18 ± 0.06
Glutamic acid + glutamine ^#^	12.85 ± 0.55	16.93 ± 0.14	14.05 ± 0.48	18.20 ± 0.65
Glycine	3.98 ± 0.01	3.91 ± 0.19	3.96 ± 0.01	3.82 ± 0.07
Proline	7.20 ± 0.19	2.00 ± 0.20	6.52 ± 0.11	6.76 ± 0.33
Serine + asparagine + OH-proline ^#^	7.78 ± 0.12	8.52 ± 0.06	6.05 ± 0.33	11.85 ± 0.33
Tyrosine	3.84 ± 0.02	4.77 ± 0.40	2.40 ± 0.10	2.62 ± 0.03
IVPD	79.3 ± 4.5	88.3 ± 3.0	85.6 ± 2.9	90.1 ± 2.5

Data correspond to the mean ± standard deviation of two replicates for the amino acid profile and of three repetitions for in vitro protein digestibility. Amino acid values are expressed as g·100 g^−1^ protein determined from samples on a dry basis. ^#^ Amino acids that co-eluted in the chromatogram.

## Data Availability

The original contributions presented in this study are included in the article. Further inquiries can be directed to the corresponding authors.
